# Spectral Clustering Algorithm Based on Improved Gaussian Kernel Function and Beetle Antennae Search with Damping Factor

**DOI:** 10.1155/2020/1648573

**Published:** 2020-05-29

**Authors:** Zhe Zhang, Xiyu Liu, Lin Wang

**Affiliations:** Business School, Shandong Normal University, Jinan, China

## Abstract

There are two problems in the traditional spectral clustering algorithm. Firstly, when it uses Gaussian kernel function to construct the similarity matrix, different scale parameters in Gaussian kernel function will lead to different results of the algorithm. Secondly, K-means algorithm is often used in the clustering stage of the spectral clustering algorithm. It needs to initialize the cluster center randomly, which will result in the instability of the results. In this paper, an improved spectral clustering algorithm is proposed to solve these two problems. In constructing a similarity matrix, we proposed an improved Gaussian kernel function, which is based on the distance information of some nearest neighbors and can adaptively select scale parameters. In the clustering stage, beetle antennae search algorithm with damping factor is proposed to complete the clustering to overcome the problem of instability of the clustering results. In the experiment, we use four artificial data sets and seven UCI data sets to verify the performance of our algorithm. In addition, four images in BSDS500 image data sets are segmented in this paper, and the results show that our algorithm is better than other comparison algorithms in image segmentation.

## 1. Introduction

Clustering analysis is an important research problem in the field of data mining. The purpose of clustering is to divide the data set into different clusters according to the intrinsic structure and relationship between the data so that the similarity between data points within the same cluster is higher, and the similarity between data points in different clusters is lower. The main clustering methods include partitioning-based clustering, hierarchical clustering, density-based clustering, grid-based clustering, and graph theory-based clustering. Different clustering algorithms are also applied to different fields, such as image segmentation [1–4], text clustering [5, 6], and community division [7–9].

Spectral clustering is a kind of clustering algorithm based on graph theory. By spectral graph partition theory [10], the clustering problem of the data set is transformed into the graph partition problem. In spectral clustering, each data point is regarded as the vertex of the graph, and the similarity between data points is regarded as the weight of the edge. By dividing the graph, the sum of the weight of the edge in the subgraph is as high as possible, and the sum of the weight of the edge between different subgraphs is as low as possible.

In 1973, Donath and Hoffman [10] first proposed the concept of graph partition based on the adjacency matrix, marking the formal birth of spectral clustering. In the same year, Fiedler [11] found that the two-way partition of the undirected graph is closely related to the eigenvector corresponding to the second small eigenvalue of the corresponding Laplacian matrix, which provides a new way to solve the problem of graph partition. In 2000, Shi and Malik [12] put forward the standard cut objective function, also known as the N-cut criterion, based on the spectral theory. In 2001, Ding et al. [13] put forward the minimum and maximum cut-set criterion based on N-cut, which balances the two requirements of minimum division loss and maximum vertex number of subgraphs, making division more inclined to balance the cut set and avoiding segmentation of smaller subgraphs with only a few vertices. In 2002, Jordan, Weiss, and Ng [14] proposed NJW algorithm, which is different from two-way division. The algorithm is based on *k*-way division, and it is also the most widely used spectral clustering algorithm so far. Despite the good development of spectral clustering, there are still some problems with the algorithm itself, such as how to select the scale parameters in the Gaussian kernel function. In 2004, scholars [15] have proved that the selection of scale parameters will affect the clustering results. To solve this problem, Zhang et al. [16] proposed a construction method of the similarity matrix based on local density. Nataliani and Yang [17] proposed an energy Gaussian kernel function to solve this problem.

Beetle antennae search algorithm (BAS) is an optimization algorithm inspired by the beetle's foraging principle proposed by Jiang and Li [18] in 2017. By simulating the detection function of beetle's tentacles and the mechanism of beetle's random walking, an optimization mechanism similar to beetle's foraging process is realized. According to the smell of food, the moving direction of the beetle is determined. When the smell of the left tentacle is strong, it will move to the left; otherwise, it will move to the right. Through the random orientation mechanism and variable step size mechanism, a beetle can search in the global scope. Compared with other intelligent algorithms, the algorithm does not need to know the specific form of gradient information and function and has the advantages of fast convergence speed and low requirements for parameters. So, it has been applied in some fields. Wang and Liu [19] combined the reverse neural network with the BAS algorithm to predict the loss of storm disaster. Chen et al. [20] used the particle swarm optimization algorithm based on the BAS algorithm to solve the portfolio model. Wang and Chen [21] proposed a kind of bee swarm antenna search algorithm (BSAS).

The main contributions of this paper are as follows: (1) A construction method of the similarity matrix is proposed, which uses the distance information of some nearest neighbors to define the scale parameter *σ* to overcome the influence of artificial designated scale parameter *σ* on the results. (2) In the clustering stage, we use the proposed beetle antennae search algorithm with damping factor (DBAS) to complete the clustering. Through such an intelligent optimization algorithm, we can overcome the impact of random initialization of the cluster center on the results when K-means is used in the traditional spectrum clustering. And the damping factor overcomes the oscillation in the iterative process and improves the stability of the algorithm.

The content of this paper is organized as follows. In Section 3, an improved spectral clustering algorithm based on the distance information of some nearest neighbors and beetle antennae search algorithm with damping factor is proposed.  Section 4 shows the performance of the algorithm through experimental analysis. The conclusion will be presented in Section 5.

## 2. Spectral Clustering and Beetle Antennae Search Algorithm

### 2.1. Spectral Clustering

The spectral clustering algorithm uses the eigenvectors of the Laplacian matrix corresponding to the data set to cluster. In the spectral clustering algorithm, firstly, an undirected graph *G*=(*V*, *E*) is constructed according to the data points. Each vertex *v*_*i*_ on the graph corresponds to a data point, and the weight *w*_*ij*_ on the edge is the similarity between the data points. In general, we use Gaussian kernel function to construct the similar matrix. Then, we can get a degree matrix *D*, *d*_*ii*_=∑_*j*=1_^*n*^*w*_*ij*_, whose main diagonal element is equal to the sum of the row elements corresponding to the similar matrix. There are usually three ways to construct the Laplacian matrix *L*: (1) denormalized Laplacian matrix, (2) normalized symmetric Laplacian matrix *L*=*I* − *D*^−1/2^*WD*^−1/2^, and (3) normalized asymmetric Laplacian matrix *L*=*D*^−1^*WD*^−1^. The eigenvector *e*_1_*e*_2_ … *e*_*k*_ corresponding to the first *k* eigenvalues of the Laplace matrix can be calculated and set *U*=[*e*_1_*e*_2_ … *e*_*k*_]. Then, a new feature matrix *F* is obtained by normalizing *U*. Each row in the feature matrix *F* is regarded as a sample, which is clustered to obtain a group of clusters *C*_1_, *C*_2_,…, *C*_*k*_. NJW algorithm [14] is the most commonly used spectral clustering algorithm. The basic step of the NJW algorithm is shown in Algorithm 1.

### 2.2. Beetle Antennae Search Algorithm (BAS)

Based on the principle of beetle's foraging, three optimization strategies can be simplified: (1) The left and right antennae of the beetle are located on both sides of the individual. (2) The ratio of the step length of each action to the distance between two antennae is a fixed constant. (3) After a move, the direction of its head is random. Then, we can build an optimization model (the beetle is simplified as an individual):

1. For an optimization problem in the *n*-dimensional space, *x*_*l*_ is used to represent the coordinates of the left antennae of an individual, *x*_*r*_ represent the coordinates of the right antennae of an individual, and *x* is the centroid coordinate. *D*_0_ is the distance between two antennae. Since the orientation of the individual is random after each movement, the direction of the vector that the right of the individual points to the left is also random. It can be expressed by a normalized random vector *di* *r* = *di* *r*/*norm*(*ran* *ds*(*n*, 1)). There is *x*_*l*_ − *x*_*r*_ = *D*_0_*∗di* *r*.
2. For the minimization objective function *f*(*x*), *f*_left_ = *f*(*x*_*l*_) and *f*_right_ = *f*(*x*_*r*_). If *f*_left_ is less than *f*_right_, then the individual travels in the direction of the left antennae step, otherwise, the distance step of the individual toward the right antennae direction.

(1) $$\begin{array}{c} x=x-\text{step}*\text{normal}\left(x_{l}-x_{r}\right)*\text{sign}\left(f_{\text{left}}-f_{\text{right}}\right). \end{array}$$

(3) Repeat step 1 and step 2 until the maximum number of iterations is reached or the individual does not change in *M* iterations.

## 3. Improved Spectral Clustering Algorithm

In this section, we improve Gaussian kernel function and BAS algorithm, respectively. After using the new Gaussian kernel function to construct the similarity matrix, we use the spectral clustering algorithm to get a new feature matrix, and then, we use the improved BAS algorithm to cluster.

### 3.1. An Improved Gaussian Kernel Function

In the traditional spectral clustering, the similarity matrix is usually constructed according to the Gaussian kernel function in the formula of Algorithm 1, where *σ* is the scale parameter; in general, the scale parameter *σ* is selected artificially. In 2004, scholars [15] had proved that the selection of scale parameters will affect the clustering results. In order to solve this problem, this paper proposes a method of constructing a similarity matrix based on the distance information of some nearest neighbors:

(2) $$\begin{array}{c} w_{ij}=\exp\left(\frac{-{\left\|x_{i}-x_{j}\right\|}^{2}}{\sigma_{i}\sigma_{j}}\right), \end{array}$$

where *σ*_*i*_=(1/*g*)∑_*j*=1_^*g*^*dist*(*x*_*i*_, *x*_*j*_), which is the mean distance of the nearest *g* points from point *i*. *g* is the ratio of the total number of samples to the square of the number of clusters. *g*=*N*/*k*^2^, where *N* is the total number of samples and *k* is the number of clusters.

### 3.2. Beetle Antennae Search Algorithm with Damping Factor (DBAS)

As mentioned in Section 2.2, the direction of the individual is random in each iteration. This results in more oscillations in the process of algorithm iteration. It is possible that the result of the *M* + 1 iteration is worse than that of the *M* iteration many times. We proposed to add a damping factor to the formula of the position update of the individual, which updates the position information by using the results of this iteration and the last iteration. The formula is described as

(3) $$\begin{array}{c} x_{t+1}=x_{t+1}*\left(1-da\;mp\right)+x_{t}*damp, \end{array}$$

where *x*_*t*_ indicates the position in the *t* − 1th iteration, *da* *mp* ∈ [0.5, 1).

**Figure 1:**
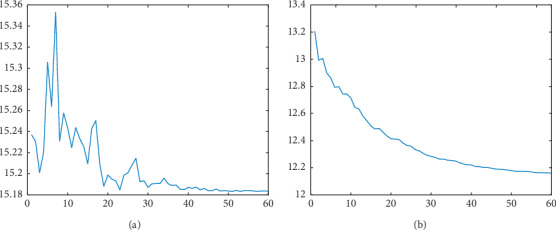
Iterative results of BAS with or without damping factor in the Iris data set. (a) BAS. (b) BAS with damping factor.

We use the algorithm with damping factor and the algorithm without damping factor to experiment on the Iris data set.  Figure 1 shows that adding damping factor to the algorithm can effectively overcome the oscillation problem in the iterative process.

### 3.3. SC-DBAS Algorithm

Firstly, we use the Gaussian kernel function based on the distance information of some nearest neighbors (formula 2) to construct the similarity matrix and then calculate the corresponding degree matrix and Laplace matrix. We select the eigenvectors corresponding to the first *k* minimum eigenvalues of the Laplace matrix to construct an eigenmatrix and then normalize it to get a new eigenmatrix. Each row of the matrix is regarded as a sample point. For such a new data set, we randomly initialize a group of cluster centers as an individual and then use DBAS algorithm to cluster. SC-DBAS algorithm flow is given in Algorithm 2.

### 3.4. Computational Complexity

The computational complexity of the proposed algorithm can be calculated as follows: the SC-DBAS algorithm is divided into three parts: (1) constructing a similar graph, which needs *O*(*n*^2^), (2) eigenvalue decomposition, which needs *O*(*n*^3^), and (3) clustering by using DBAS algorithm, which needs *O*(*nkl*), where *k* is the number of cluster centers and *l* is the number of iterations. According to the notation of big *O*, the computational complexity of the proposed algorithm is *O*(*n*^3^).

## 4. Experimental Results and Analysis

### 4.1. Experimental Setting

All the experiments are conducted on the computer with Intel core i5-3230M CPU, 8 GB RAM. The experiment environment is Matlab 2016b. In the experiment, we compare the proposed algorithm with the K-means, NJW [14], MPSC algorithm [22], PGSC algorithm [17], and SC-NP algorithm [23] on four artificial data sets and seven UCI data sets. The proposed algorithm will also use the image in the BSDS500 data set for image segmentation. In the experimental part of image segmentation, the comparison algorithm is K-means, NJW [14], PGSC algorithm [17], and SC-NP algorithm [23].

In the experiment, the parameters are set as follows: step = 0.1; step adjustment factor eta = 0.95; the ratio between step and *D*_0_ is 5; the number of iterations *n* = 100; and damp = 0.5. The information of data sets is shown in Table 1.

### 4.2. Evaluation Indicators

In the experiment, we use four indicators to evaluate the clustering results: accuracy, ARI, F1 score, and time (s).

1. Accuracy rate: the accuracy rate represents the proportion of the number of correct clustering samples to the total number of samples, where *V* is the division label and *U* is the real label:

(4) $$\begin{array}{c} Acc=\frac{1}{N}\sum_{i=1}^{k}\underset{j}{\max}\left|v_{i}\;\cap \;u_{j}\right|, \end{array}$$

(2) ARI: there are four cases by comparing the calculation results *V* with the real label *U*. SS contains sample pairs that belong to the same cluster in *V* and the same cluster in *U*. SD contains sample pairs that belong to the same cluster in *V* but not the same cluster in *U*. DS contains sample pairs that do not belong to the same cluster in *V* but belong to the same cluster in *U*. DD contains sample pairs that do not belong to the same cluster in *V* and do not belong to the same cluster in *U*. Set *SS*=*a*, *S* *D*=*b*, *DS*=*c*, *D* *D* =*d*; there are

(5) $$\begin{array}{c} \text{ARI}=\frac{2\left(a\;d-bc\right)}{\left(a+b\right)\left(b+d\right)+\left(a+c\right)\left(c+d\right)}. \end{array}$$

The larger the value of ARI means that the clustering results are more consistent with the real situation.

(3) F1 score: F1 score is one of the commonly used evaluation criteria in information retrieval. It is a weighted harmonic mean value based on precision and recall. Its definition is as follows, where *a*, *b*, and *c* have been defined in the above content:

(6) $$\begin{array}{c} F1=\frac{2*P*R}{P+R},\\ P=\frac{a}{a+b},\\ R=\frac{a}{a+c}. \end{array}$$

(4) Time: in this paper, we use the average time of each algorithm running 100 times as the evaluation index.

### 4.3. Data Set Experiment Result Analysis

#### 4.3.1. Experimental Results of Artificial Data Sets

**Figure 2:**
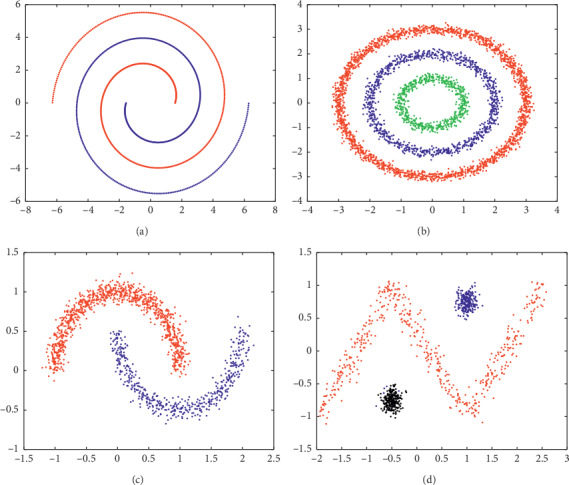
Clustering results using the proposed algorithm for artificial data sets. (a) Spiral. (b) Three circles. (c) Two moons. (d) Zigzag.

Table 2 shows the experimental results of the six algorithms on the four artificial data sets. From Figure 2, we can see that our proposed algorithm can well divide the data sets of various structures.

#### 4.3.2. Experimental Results of UCI Data Sets

**Figure 3:**
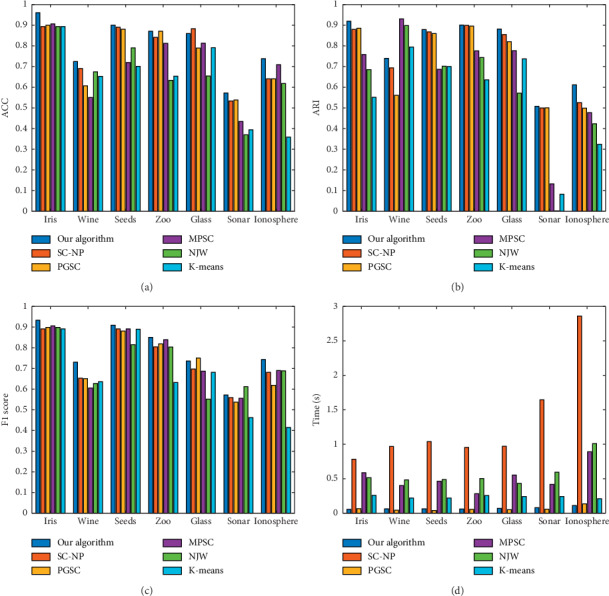
Results of six algorithms on UCI data sets. (a) Accuracy. (b) ARI. (c) F1 score. (d) Time (s).

Table 3 and Figure 3 show the experimental results of the six algorithms on seven UCI data sets. By comparing the results, we can see that the algorithm proposed in this paper performs better than the other five algorithms and has a shorter running time.

### 4.4. Application of the SC-DBAS Algorithm to Image Segmentation

Clustering-based image segmentation is based on the similarity between image pixels; through some clustering algorithms, the pixels are divided into different clusters so as to complete the segmentation of the original image.

**Figure 4:**
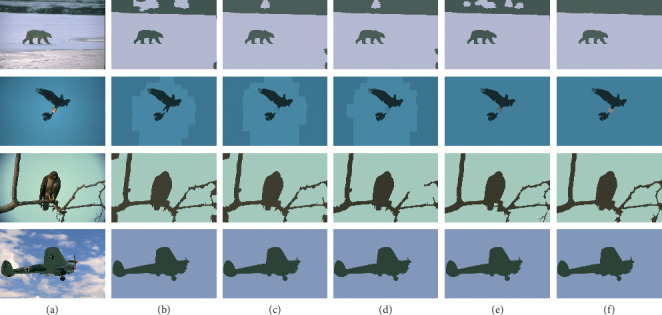
(a) Original image, (b) K-means, (c) NJW, (d) PGSC, (e) SC-NP, and (f) our proposed algorithm.

In this section, we segment some images of the BSDS500 data set. For a 481∗321 pixels image, if we treat each pixel as a data point, there will be 154,401 data points. Therefore, in order to reduce the scale of data points, we first use SLIC algorithm [24] to perform presegmentation (superpixel segmentation) on the image. Each superpixel is an oversegmented region and is considered as a data point. Then, the proposed algorithm is used to segment the image. In the experiment, the number of superpixels of each image is 200. The comparison algorithm used in the experiment is K-means, NJW [14], PGSC algorithm [17], and SC-NP algorithm [23]. Then, we can get the results which are given in Figure 4.

From the experimental results, we can see that our algorithm can segment the object and the background better, while the other four comparison algorithms will have the wrong segmentation area. The segmentation accuracy results are shown in Table 4.

## 5. Conclusion

In this paper, an improved spectral clustering algorithm combined with the improved BAS algorithm is proposed. The proposed algorithm first improves the construction of the similarity matrix, which uses the distance information of some nearest neighbors of each point to calculate the corresponding scale parameters. In the stage of clustering, we proposed BAS algorithm with damping factor to cluster, which can overcome the problem that the original algorithm oscillates many times in the iterative process. The experimental results show that our algorithm is better than other algorithms in UCI data sets, artificial data sets, and image segmentation. However, in the application of image segmentation, our results will be affected by the effect of superpixel segmentation. The future work is to improve our algorithm so that it does not need to preprocess in image segmentation and can directly segment the image, and we will use more real images and medical images to verify our algorithm.

## Figures and Tables

**Algorithm 1:**
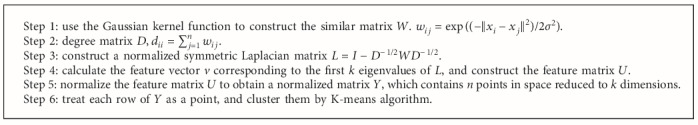
NJW algorithm.

**Algorithm 2:**
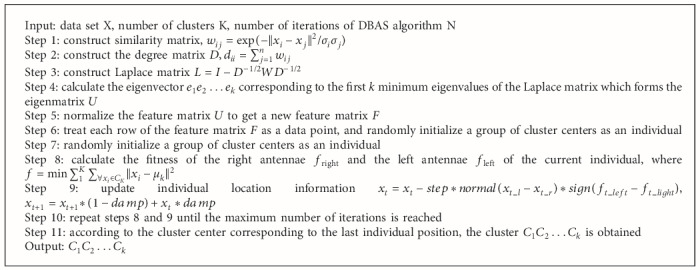
SC-DBAS algorithm.

**Table 1:** Data set information.

Data set	Objects	Attributes	Classes	Source
Iris	150	4	3	UCI
Wine	178	13	3	UCI
Seeds	210	6	3	UCI
Zoo	101	16	7	UCI
Glass	214	10	6	UCI
Sonar	208	60	2	UCI
Ionosphere	351	34	2	UCI
Spiral	944	2	2	Artificial
Two moons	2000	2	2	Artificial
Three circles	3603	2	3	Artificial
Zigzag	1002	2	3	Artificial

**Table 2:** Accuracy of six algorithms on artificial data sets.

Data set	K-means	NJW	MPSC	PGSC	SC-NP	SC-DBAS
Spiral	0.5975	1	1	1	0.5890	1
Two moons	0.7337	1	1	1	0.7170	1
Three circles	0.5554	1	1	1	0.5753	1
Zigzag	0.7076	1	1	1	0.7275	1

**Table 3:** Results of six algorithms on UCI data sets.

Data set	Evaluation indicators	K-means	NJW	MPSC	PGSC	SC-NP	SC-DBAS
Iris	Accuracy	0.8933	0.8933	0.9067	0.9000	0.8933	**0.9600**
	ARI	0.5516	0.6850	0.7583	0.8859	0.8797	**0.9195**
	F1 score	0.8918	0.8988	0.9057	0.8988	0.8918	**0.9332**
	Time (s)	0.2610	0.5164	0.5880	0.0669	0.7841	0.0553

Wine	Accuracy	0.6530	0.6742	0.5505	0.6067	0.6910	**0.7247**
	ARI	0.7943	0.8986	0.9310	0.5614	0.6938	**0.7395**
	F1 score	0.6363	0.6276	0.6057	0.6510	0.6531	**0.7302**
	Time (s)	0.2206	0.4845	0.4020	0.0443	0.9687	0.0638

Seeds	Accuracy	0.7008	0.7905	0.7194	0.8810	0.8905	**0.9000**
	ARI	0.7006	0.7022	0.6865	0.8594	0.8681	**0.8787**
	F1 score	0.8897	0.8150	0.8914	0.8813	0.8913	**0.9092**
	Time (s)	0.2195	0.4893	0.4641	0.0403	1.0381	0.0641

Zoo	Accuracy	0.6534	0.6337	0.8119	**0.8713**	0.8416	**0.8713**
	ARI	0.6359	0.7441	0.7758	0.8962	0.8994	**0.9012**
	F1 score	0.6319	0.8038	0.8389	0.8190	0.8045	**0.8540**
	Time (s)	0.2561	0.5035	0.2844	0.0555	0.9538	0.0608

Glass	Accuracy	0.7913	0.6542	0.8131	0.7897	**0.8832**	0.8598
	ARI	0.7375	0.5718	0.7767	0.8206	0.8552	**0.8817**
	F1 score	0.6812	0.5515	0.6872	**0.7509**	0.6973	0.7364
	Time (s)	0.2418	0.4335	0.5547	0.0512	0.9710	0.0712

Sonar	Accuracy	0.3942	0.3701	0.4346	0.5385	0.5337	**0.5721**
	ARI	0.0827	0.0022	0.1324	0.5006	0.4999	**0.5080**
	F1 score	0.4623	0.6119	0.5556	0.5370	0.5593	**0.5716**
	Time (s)	0.2422	0.5950	0.4181	0.0587	1.6458	0.0794

Ionosphere	Accuracy	0.3589	0.6182	0.7094	0.6410	0.6410	**0.7379**
	ARI	0.3240	0.4237	0.4772	0.4989	0.5253	**0.6121**
	F1 score	0.4149	0.6885	0.6902	0.6188	0.6817	**0.7431**
	Time (s)	0.2105	1.011	0.892	0.1354	2.8606	0.1108

**Table 4:** Accuracy of five algorithms on images.

	K-means	NJW	PGSC	SC-NP	SC-DBAS
Image 1	0.8804	0.9483	0.9483	0.9304	0.9943
Image 2	0.5562	0.5562	0.5347	0.9942	0.9969
Image 3	0.9520	0.9696	0.9729	0.9722	0.9741
Image 4	0.9902	0.9883	0.9920	0.9917	0.9924

## Data Availability

The four artificial data sets that were manually generated can be obtained by contacting the author. The seven UCI data sets are often used in the existing literature which are from the UCI Machine Learning Repository available at http://archive.ics.uci.edu/ml/datasets.php. The four tested images are from the Berkeley computer vision group, Berkeley segmentation data set, and benchmark 500 (BSDS500), which are available at https://www2.eecs.berkeley.edu/Research/Projects/CS/vision/grouping/resources.html.
